# Mediterranean diet and brain functional connectivity in a population without dementia

**DOI:** 10.3389/fnimg.2024.1473399

**Published:** 2024-12-06

**Authors:** Efstratios Karavasilis, Vasileios Balomenos, Foteini Christidi, Georgios Velonakis, Georgia Angelopoulou, Mary Yannakoulia, Eirini Mamalaki, Archontoula Drouka, Dora Brikou, Angeliki Tsapanou, Yian Gu, Nikolaos Scarmeas

**Affiliations:** ^1^Medical Physics Lab, Scholl of Medicine, Democritus University of Thrace, Alexandroupolis, Greece; ^2^Department of Imaging and Interventional Radiology, ‘Sotiria' General and Chest Diseases Hospital of Athens, Athens, Greece; ^3^Research Unit of Radiology and Medical Imaging, 2nd Department of Radiology, Attikon General University Hospital, School of Medicine, National and Kapodistrian University of Athens, Athens, Greece; ^4^First Department of Neurology, Aiginition Hospital, School of Medicine, National and Kapodistrian University of Athens, Athens, Greece; ^5^Computational Neuroimaging Group (CNG), School of Medicine, Trinity College Dublin, Dublin, Ireland; ^6^Department of Nutrition and Dietetics, Harokopio University, Athens, Greece; ^7^Taub Institute for Research in Alzheimer's Disease and the Aging Brain, The Gertrude H. Sergievsky Center, Columbia University, New York, NY, United States

**Keywords:** mediterranean diet, rs-fMRI, functional connectivity, aging, biomarkers

## Abstract

**Introduction:**

Adjustable lifestyle factors, such as diet, are associated with cognitive functions, structural and functional brain measures, but the association between the functional connectivity (FC) and the Mediterranean Diet (Medicine) in population without dementia is yet to be explored.

**Methods:**

The association between MeDi and brain FC in 105 individuals without dementia aged 63 (SD ± 8.72) years old who underwent brain MRI including resting-state (rs) functional MRI (fMRI) was examined. Dietary intake was evaluated through four 24-h recalls using the multiple-pass method and adherence to the MeDi was estimated using the MedDietScore, with higher values indicating greater adherence to MeDi. Multivariable linear regression models were used to investigate the associations between FC (both positive and negative associations) and MedDietScore.

**Results:**

Rs-fMRI analysis revealed significant associations between FC and MedDietScore. The FC between the medial prefrontal cortex and a cluster located in left postcentral gyrus and in the left supramarginal gyrus was positively associated with MedDietScore. On the other hand, the FC between medial visual and right posterior division of both middle and superior temporal gyrus was negatively associated with MedDietScore. Of note, a temporal negative correlation was detected between above-mentioned FC networks. The FC between superior temporal gyrus and occipital regions was associated with participants' attention, executive functions, and memory scores. Furthermore, the associations for attention and executive functions were pronounced in participants with high adherence to MeDi compared to those with low adherence to MeDi.

**Discussion:**

In conclusion, our study documented an association between higher adherence to MeDi and rs-FC in fronto-parietal and temporo-occipital regions, particularly in areas that are involved in cognitive processes altered across normal and pathological aging. From a clinical point of view, our findings support a favorable role of MeDi on FC which may have significant clinical implications in the rapidly aging population. Rs-fMRI is also proposed as a useful tool in the emerging field of nutritional neuroscience and a candidate non-invasive biomarker of brain aging.

## 1 Introduction

Mediterranean diet (MeDi), is a traditional healthy dietary pattern, characterized by a plethora of plant foods (fruits, vegetables, cereals, legumes, nuts, and seeds), olive oil as the principal source of fat, moderate consumption of dairy products (principally cheese and yogurt), regular consumption of fish (particularly fish rich in omega-3 fatty acids) and poultry, low consumption of red meat and low-to-moderate consumption of wine. Adherence to MeDi is often evaluated through diet scores, with high scores often indicating high adherence to this dietary pattern. MeDi has been associated with decreased risk of Alzheimer's disease (AD) and mild cognitive impairment (MCI) as well as delayed cognitive decline (Scarmeas et al., 2006; Gu et al., 2015; Ballarini et al., 2021). MeDi is also considered to play an important role in brain structural integrity (Drouka et al., 2022). However, the available studies investigating the association between MeDi and brain imaging biomarkers are limited (Drouka et al., 2022; Townsend et al., 2022). The majority of available studies support the role of higher adherence to MeDi mainly focusing on cortical thickness and brain volume (Mosconi et al., 2014; Gu et al., 2015; Luciano et al., 2017; Staubo et al., 2017; Croll et al., 2018; Karstens et al., 2019), white matter integrity (Pelletier et al., 2015; Christidi et al., 2024), white matter hyperintensities, beta amyloid accumulation and burden (Gu et al., 2015, 2016; Rodrigues et al., 2020).

Resting-state fMRI (rsfMRI) is an imaging method, based on the blood-oxygen-level-dependent (BOLD) signal, which examines brain networks when no task is performed and estimates functional associations between different regions. The latter may be functionally related based on their synchronous activation, which is called “functional connectivity” (FC). Several studies have highlighted age as a contributing factor to the reduced FC within the rsfMRI networks such as default mode network (DMN) or less specific between different functional nodes and those changes can be observed by early middle age (Geerligs et al., 2014; Varangis et al., 2019a,b; He et al., 2020; Malagurski et al., 2020). Decreased cognitive network segregation is correlated with poorer cognitive performance (Cassady et al., 2019; Ng et al., 2016; Sato et al., 2024) supporting the suggestion of topology in rsfMRI is critical in coordinating as well as in transferring information in cognitive task processes.

Diet is one of the modifiable lifestyle factors that could essentially refine FC patterns related to cognitive function. However, its role remains unclear. To the best of our knowledge only two studies have investigated the role of MeDi in brain FC cross-sectionally (Gaynor et al., 2022b) and longitudinally (Gaynor et al., 2022a). The authors did not find any correlation between MeDi and overall inter-network FC in their main cross-sectional analysis (Gaynor et al., 2022b). However, a significant moderation role of MeDi on the association between rsFC and cognition was found, with the association between overall rsFC and fluid reasoning score being weaker in the moderate MeDi group and high MeDi group than in the low (Gaynor et al., 2022b). The longitudinal study highlighted that MeDi may protect cognitive function by attenuating the negative effects of changes in connectivity over time (Gaynor et al., 2022a). It should be noted that both studies focused only on positive inter-network correlations, excluding a priori all negative correlations (Gaynor et al., 2022a,b).

Thus, the aim of this study was to investigate the association between MeDiScore and brain FC in community-dwelling individuals without dementia, analyzing both positive and negative functional correlations, with the negative ones indicating a negative association between the spontaneous BOLD signals of two FC brain regions. According to previous MeDi-related neuroimaging studies, we hypothesize that higher adherence to MeDi will be associated with specific patterns of FC between brain areas that are implicated in aging.

## 2 Material and methods

### 2.1 Participants

Aiginition Longitudinal Biomarker Investigation of Neurodegeneration (ALBION) is a longitudinal study taking place in the Cognitive Disorders Clinic of Aiginition Hospital of the National and Kapodistrian University of Athens, and is designed to address research questions regarding the preclinical and prodromal stages of AD. A detailed description of the study protocol has been published previously (Kalligerou et al., 2019; Scarmeas et al., 2022). Briefly, study participants include people aged 40 years or older visiting the cognitive disorders' outpatient clinic of a tertiary university hospital. These participants may have concerns about their cognitive status or may be asymptomatic but committed to contributing to medical science. Exclusion criteria are diagnosis of dementia, neurological, psychiatric or medical conditions associated with a high risk of cognitive impairment or dementia, MRI contraindications, as well as the use of anticoagulant medication.

### 2.2 Cognitive assessment

Global cognitive status was assessed using the Mini Mental State Examination (MMSE) (Folstein et al., 1975) and the Addrenbrooke's Cognitive Examination-revised (ACE-R) (Mioshi et al., 2006), while the pre-morbid level was estimated based on vocabulary. Attention, executive function, memory, language and visuospatial cognitive domains were examined from different neuropsychological tests. A detailed description is presented elsewhere (Brikou et al., 2023). Participants' raw scores on the individual neuropsychological tests for each cognitive domain were transformed to *z*-scores using mean and standard deviation values derived from the healthy controls of the study sample. An average domain score for each cognitive domain (i.e. attention, executive functions, memory, language and visual-spatial functioning) was produced (with a higher score indicating better cognitive performance). All tests were administered by trained neuropsychologists.

In the analyses of the current study, 120 volunteers without dementia who had full dietary and MRI data at baseline (first visit evaluation) were included.

### 2.3 Dietary assessment

Dietary intake was evaluated through four 24-h recalls using the multiple-pass method (Conway et al., 2004). Participants were interviewed by appropriately trained registered dietitians, and they were asked to report in detail all foods and beverages consumed the day before. The first recall was conducted in person and the subsequent ones over the phone. Three of the recalls were conducted once per week for the next 3 weeks, on weekdays and one on a weekend day to more accurately estimate usual intake throughout the week. Participants were not aware of the day of the recall in advance, so they could not change their diet in anticipation of the interview. Recall data were grouped into specific food groups, namely full- and low- fat dairy products, non-refined cereals (whole bread, pasta, rice, other grain), fruits, vegetables, potatoes, red meat and products, poultry, fish, legumes, added fats, alcoholic beverages, sweets etc.

Based on this food grouping, adherence to the MeDi was estimated using the MedDietScore (Panagiotakos et al., 2006). The MedDietScore was based on the weekly consumption of 11 food groups. For the food groups that are presumed to closely characterize the Mediterranean pattern (i.e., non-refined cereals, fruits, vegetables, legumes, potatoes, fish and olive oil), individuals who reported no consumption were assigned a score of 0, and scores of 1–5 are assigned for rare to daily consumption. For those foods that are presumed to diverge from this diet pattern (i.e., meat and meat products, poultry and full-fat dairy products), participants were assigned scores on a reverse scale. For alcohol intake, a score of 5 was assigned for consumption of <300 ml of alcohol/day, a score of 0 was assigned for no consumption or for consumption of 700 ml/day and scores of 4–1 were assigned for consumption of 600–700, 500–600, 400–500, and 300–400 mL/day, respectively. Total MedDietScore ranges from 0 to 55, with higher MedDietScore values indicating greater adherence to the MeDi pattern.

### 2.4 Imaging data acquisition

All participants underwent a brain MRI on a 3T Achieva TX Philips manufactured MRI scanner (Philips, Best, the Netherlands) equipped with an eight-channel head coil using the same imaging protocol. The imaging protocol included an anatomical high resolution 3D high T1 (3D-HR-T1) weighted sequence (repetition time (TR): 6.7 ms, time echo (TE): 3.1 ms, flip angle: 9, voxel size 1.1 × 1.1 × 1.2 mm^3^, sagittal orientation), a whole-brain functional T2^*^ weighted gradient echo combined with echo planar imaging (TR: 1,900 ms, TE: 30 ms, flip angle: 90°, acquisition voxel size 3.3 × 3.3 × 3.3 mm^3^ and sensitivity encoding reduction factor of two, 204 dynamic scans ~ 6 min 34 s), as well as clinically used imaging techniques such as 2D T2 turbo spin echo and 2D T2 weighted with fluid attenuated inversion recovery (FLAIR) sequence. Foam pads were positioned to participants' head to minimize voluntary motion and the participants were instructed to lie still with their eyes closed. T2, 3D T1 and T2 Flair images were reviewed by an experienced neuroradiologist (G.V.) for unexpected findings whereas fMRI data were reviewed by an MR physicist (E.K.) for motion or scanner related artifacts. Imaging data of 105 participants (out of 120) were finally analyzed due to the presence of motion artifacts and unexpected findings in 10 and five participants, respectively. Motion artifacts were identified by an MR physicist with >15 years of experience in neuroimaging data acquisition and analysis (EK) whereas unexpected findings were identified by an experience neuroradiologist (GV).

### 2.5 Resting state-fMRI data analyses

Rs-fMRI imaging data were pre- and post-processed using the Functional Connectivity (CONN) toolbox v22 of Matlab (http://www.nitrc.org/projects/conn).

#### 2.5.1 Preprocessing of fMRI data

All functional and anatomical 3D-High Resolution (HR) -T1 weighted images were firstly preprocessed using Statistical Parametric Mapping 12 (SPM12) that is integrated in the CONN toolbox. Functional data were realigned and unwrapped to co-register and resample all the dynamics to the middle dynamic scan using b-spline interpolation (Andersson et al., 2001). Slice time correction was performed using a sinc-interpolation considering the interleaved way of scanning. Computational imaging data quality control was performed, identifying outlier scans with displacement of 1.00 mm and blood-oxygen-level-dependent (BOLD) signal change above five standard deviations. Both structural and functional data were segmented and normalized into the Montreal Neurological Institute (MNI) space using SPM12 segmentation procedure, and they were smoothed using an 8 mm Gaussian kernel (Ashburner and Friston, 2005).

The pre-processed functional data were denoized applying linear regression of potential confounding effects in BOLD signal and frequency filtering (0.01–0.1 Hz) to remove unwanted source of signals derived from motion, respiratory, heart or other human or scanner related causes. Linear detrending and despiking were implemented before and band-pass filtering after the regression to decrease spurious signal correlations between the included anatomical regions. The estimated from preprocessing steps motion time series and their first temporal derivatives, cerebrospinal fluid, global white matter time series signal and scrubbing confounds were assumed as regressors (Friston et al., 1996; Behzadi et al., 2007; Power et al., 2014).

#### 2.5.2 Functional connectivity analyses

For FC analyses, CONN default ROIs derived from an atlas of cortical and subcortical areas from the FSL Harvard-Oxford Atlas, the cerebellar regions from the AAL atlas and the areas constituting the commonly used rs-fMRI networks: Default Mode Network (DMN), Salience Network, Dorsal Attention Network, Language Network, Visual Network, Sensorimotor Network, Cerebellar Network (CERN) were used. In our first level analysis we performed a whole-brain analysis investigating the FC of each of the available ROIs (each of them was used as seed) with the rest of the brain (seed to voxel analysis) without any a priori hypothesis. For each voxel in the brain, the Pearson's correlation coefficient was computed between the time series of the seed region and the time series of each voxel. To ensure that the correlation values were normally distributed, the CONN toolbox applies a Fisher *r*- to -*z* transformation to the FC coefficients. Both positive and negative correlation coefficients were considered, with the negative ones describing spontaneous BOLD signals in two brain regions that have a negative association, often referred as “anti-correlation”. In case of significant associations between two regions (either positive or negative association), a connectivity index (CI) was calculated for further analysis.

### 2.6 Statistical analysis

Demographic, clinical, cognitive and dietary data are presented with descriptive values. For the purpose of the main aim of the study, a linear regression model was applied to investigate any association between MeDi score and FC values between different brain anatomical areas using age, sex, education, and total daily calorie intake as nuisance parameters. For these neuroimaging analyses conducted CONN toolbox v22 of Matlab, we used a voxel threshold *p* < 0.001 uncorrected and a cluster threshold *p* < 0.05 family-wise error (FWE) – corrected to account for Type I errors (Woo et al., 2014). As a supplementary exploratory analysis, we further examined whether any resulted FC were associated with participants' cognitive performance, using linear regression models (with cognitive measures as dependent variables and MedDietScore, functional connections related to MedDietScore, and their interactions as predictors). In case the final model was significant, we considered any significant independent variables that contributed to the model whereas we further ran *post-hoc* analysis for interactions. For this exploratory analysis conducted using IBM SPSS v. 29.0, the level of statistical significance was set at *p* < 0.05.

## 3 Results

### 3.1 Participants characteristics

After exclusion of participants with motion artifacts (*n* = 10) and unexpected MRI findings (*n* = 5), the final sample consisted of 105 participants without dementia. The participants who were excluded from the study did not differ from the 105 participants in terms of age, sex, education, composite cognitive *z*-score, and total daily calorie intake. The group demographic, clinical and dietary data are summarized in Table 1. The scores of 11 food groups are presented in Supplementary Table 1.

**Table 1:** Descriptive values for the main demographic, cognitive and dietary data of the group.

**Group characteristics**	**Mean ±SD**
Age (years)	63.25 ± 8.72
Sex (F/M)	72/33
Education (years)	13.80 ± 3.76
ACE-R	88.61 ± 10.03
MMSE	27.65 ± 2.34
MedDietScore	30.33 ± 6.10
Attention *z*-score	−0.38 ± 1.27
Executive functions *z*-score	−0.16 ± 0.85
Memory *z*-score	−0.30 ± 1.15
Language *z*-score	−0.26 ± 1.14
Visuo-spatial functioning *z*-score	**-**0.20 ± 1.47

Descriptive values on sex are presented as absolute values whereas descriptive values on other variables are presented as mean ± standard deviation. F/M, female/male; MMSE, Mini-Mental State Examination; MedDietScore, Mediterranean diet total score; ACE-R, Addenbrooke's Cognitive Examination-revised total score; SD, standard deviation. Participants' raw scores on the individual neuropsychological tests for each cognitive domain (presented in detail elsewhere) were transformed to *z*-scores using mean and standard deviation values derived from the healthy controls of the study sample; higher z-scores indicate better cognitive performance.

### 3.2 Associations between functional connectivity and MeDi

FC analysis revealed significant associations between the MedDietScore and the FC of several anatomical areas (Table 2).

**Table 2:** Correlations between functional connectivity and MedDietScore.

**Seed region**	**Cluster (*x*, *y*, *z*)**	**Anatomical landmarks**	**Cluster size**	**p-FWE**	***T*-value**	***R* ^2^**
Medial prefrontal cortex	−60, −20, +20	L PostCG, L aSMG	230	0.013	4.71	0.25
Medial visual	+62, −30, −02	R pMTG, R pSTG	186	0.046	−4.39	0.22
R pSTG	+10, −92, +22	R OP	223	0.023	−4.36	0.20

L, left; R, right; PostCG, postcentral gyrus left; aSMG, anterior division supramarginal gyrus; pMTG, posterior division middle temporal gyrus; pSTG, posterior division superior temporal gyrus; OP, occipital pole. Cluster size corresponds to the number of voxels for each significant cluster. The sign of *T*-values indicates the direction of the association between the MedDietScore and the FC of the two regions, i.e., a positive sign indicates that the higher the MedDietScore the, the higher the negative FC between two regions, whereas a negative sign indicates that the higher the MedDietScore the lower the negative FC between two regions. *R*^2^ indicates the proportion of variance in functional connectivity explained by the MedDietScore.

**Figure 1:**
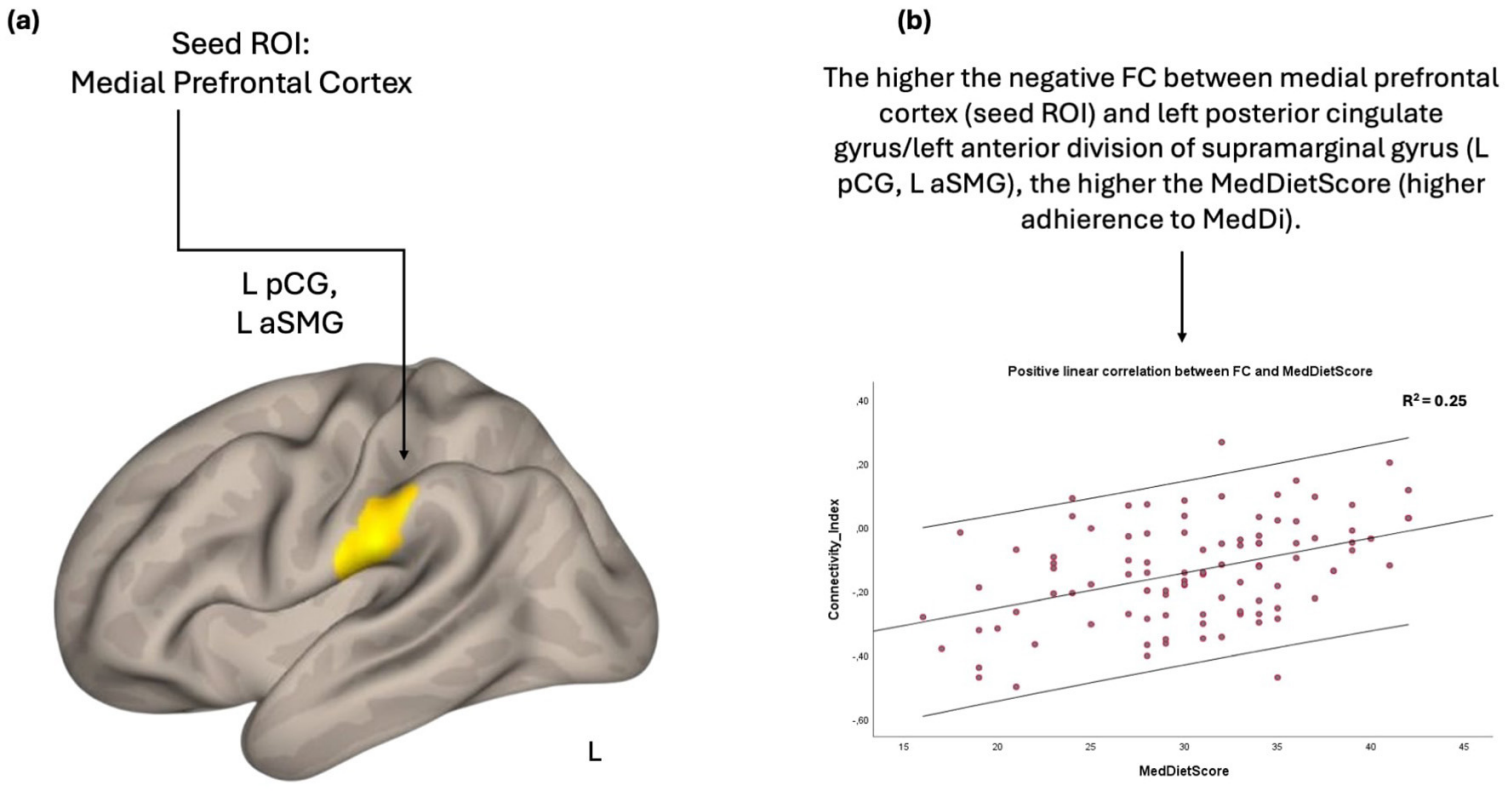
Anatomical regions (left postcentral gyrus, left anterior division of supramarginal gyrus) whose negative functional connectivity to the medial prefrontal cortex **(A)** positively correlates with MedDietScore **(B)**. Connectivity index indicates the correlation between the BOLD signal of seed region (medial prefrontal cortex) and the BOLD signal of the left postcentral gyrus, left anterior division of supramarginal gyrus. *R*^2^ indicates the proportion of variance in FC explained by the MedDietScore. ROI, region of interest; FC, functional connectivity; L, left; pCG, posterior cingulate gyrus; aSMG, anterior division of supramarginal gyrus; MedDietScore, Mediterranean Diet Score; MeDi, Mediterranean Diet.

The FC between the medial prefrontal cortex and a cluster located in left postcentral gyrus and in the left anterior division of supramarginal gyrus was positively associated with the MedDietScore. Further inspection of the connectivity values revealed that the negative FC between these anatomical regions was weakened as the adherence to MeDi increased (Figure 1).

**Figure 2:**
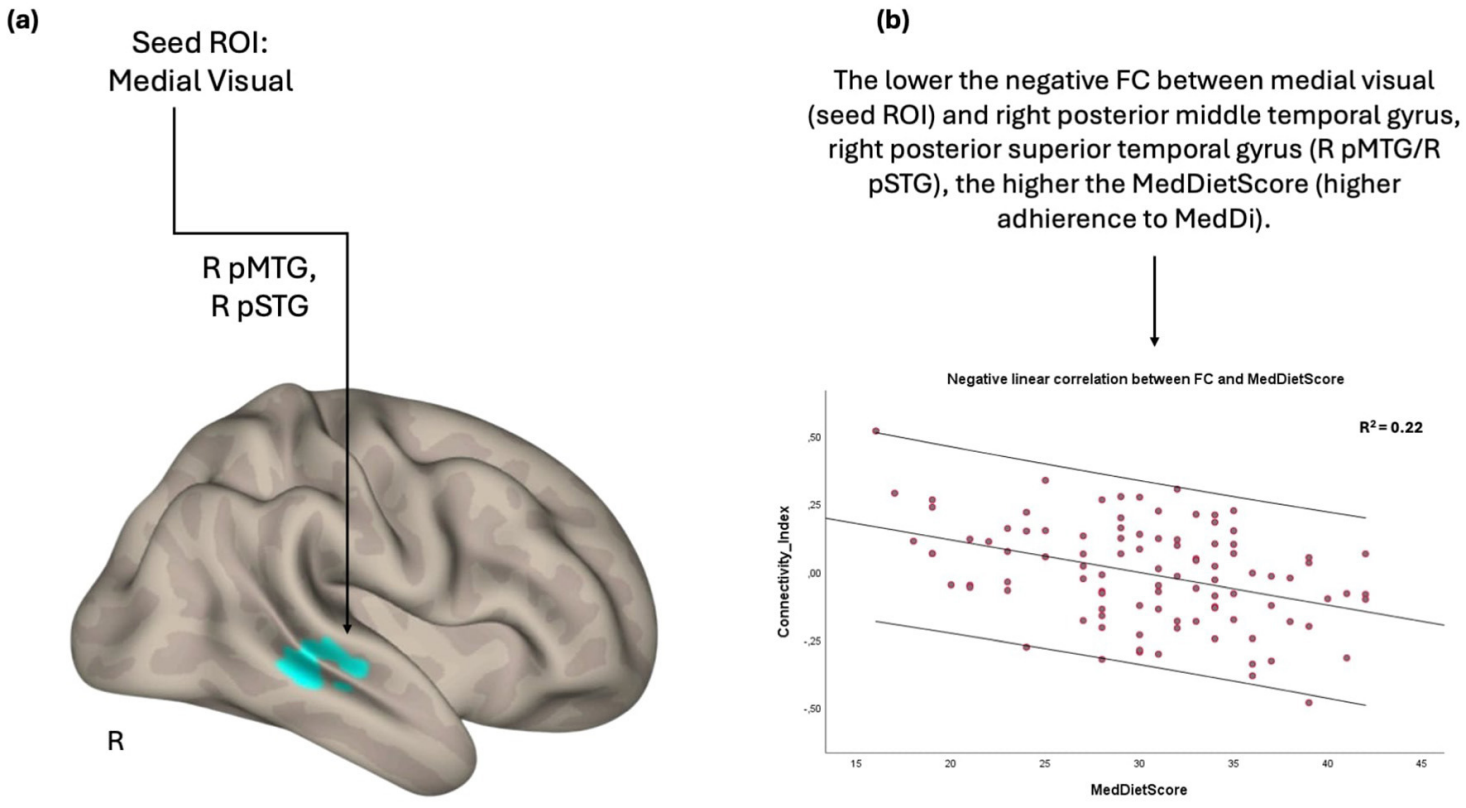
Anatomical regions (right posterior middle temporal gyrus, right posterior superior temporal gyrus) whose negative functional connectivity to the medial visual cortex **(A)** negatively correlates with MedDietScore **(B)**. Connectivity index indicates the correlation between the BOLD signal of seed region (medial visual cortex) and the BOLD signal of the right postserior middle temporal gyrus, right posterior superior temporal gyrus. *R*^2^ indicates the proportion of variance in FC explained by the MedDietScore. ROI, region of interest; FC, functional connectivity; R, right; pMTG, posterior middle temporal gyrus; pSTG, posterior superior temporal gyrus; MedDietScore, Mediterranean Diet Score; MeDi, Mediterranean Diet.

On the other hand, the FC between the medial visual and the right posterior division of both middle and superior temporal gyrus was negatively associated with the MedDietScore. Specifically, the negative FC between these anatomical regions was strengthened as the adherence to MeDi increased (Figure 2).

**Figure 3:**
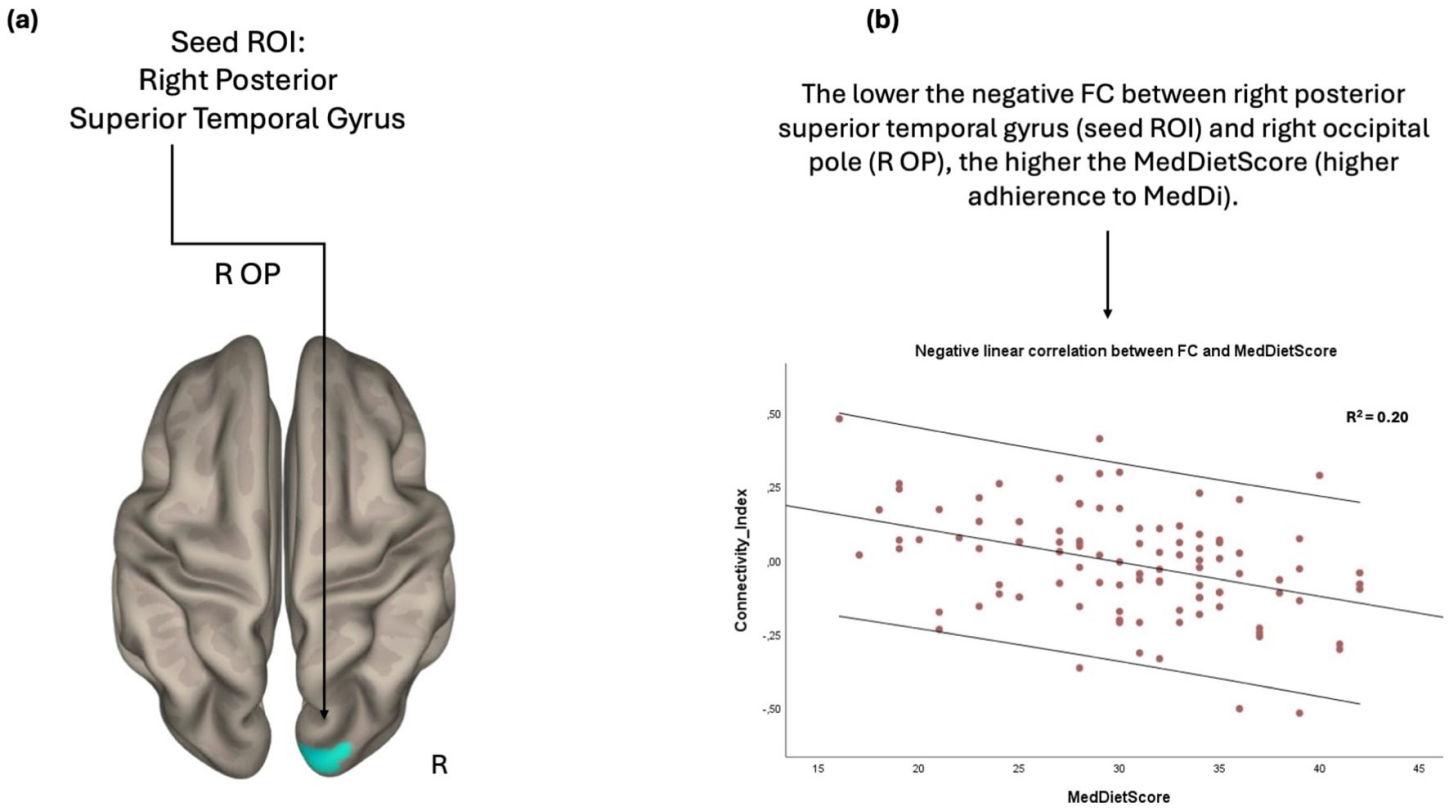
Anatomical regions (right occipital pole) whose negative FC to the right posterior superior temporal gyrus **(A)** negatively correlates with MedDietScore **(B)**. Connectivity index indicates the correlation between the BOLD signal of seed region (right posterior superior temporal gyrus) and the BOLD signal of the right occipital pole. *R*^2^ indicates the proportion of variance in FC explained by the MedDietScore. ROI, region of interest; FC, functional connectivity; R, right; OP, occipital pole; MedDietScore, Mediterranean Diet Score; MeDi, Mediterranean Diet.

Furthermore, the FC between the right posterior segment of superior temporal gyrus and right occipital lobe was inversely related to the MedDietSore; the negative FC between these anatomical regions was strengthened as the adherence to MedDietSore increased (Figure 3).

### 3.3 Associations between functional connections related to MeDi and cognitive outcomes

The functional connections related to MeDi were significantly associated with attention, executive functions, and memory. Specifically, the functional connection between right posterior superior temporal gyrus and right occipital pole was associated with attention z-score (B = −1.474, *p* = 0.037), executive functions z-score (B = −1.156, *p* = 0.015), and memory z-score (B = −1.533, *p* = 0.018); the stronger the anticorrelation between these two anatomical regions the higher the cognitive performance. No other associations between functional connections related to MeDi and cognitive scores were significant at *p* < 0.05. Of note, the MedDietScore was not related individual cognitive domains (*p* > 0.05).

### 3.4 Interaction between functional connections and MeDi on cognition

**Figure 4:**
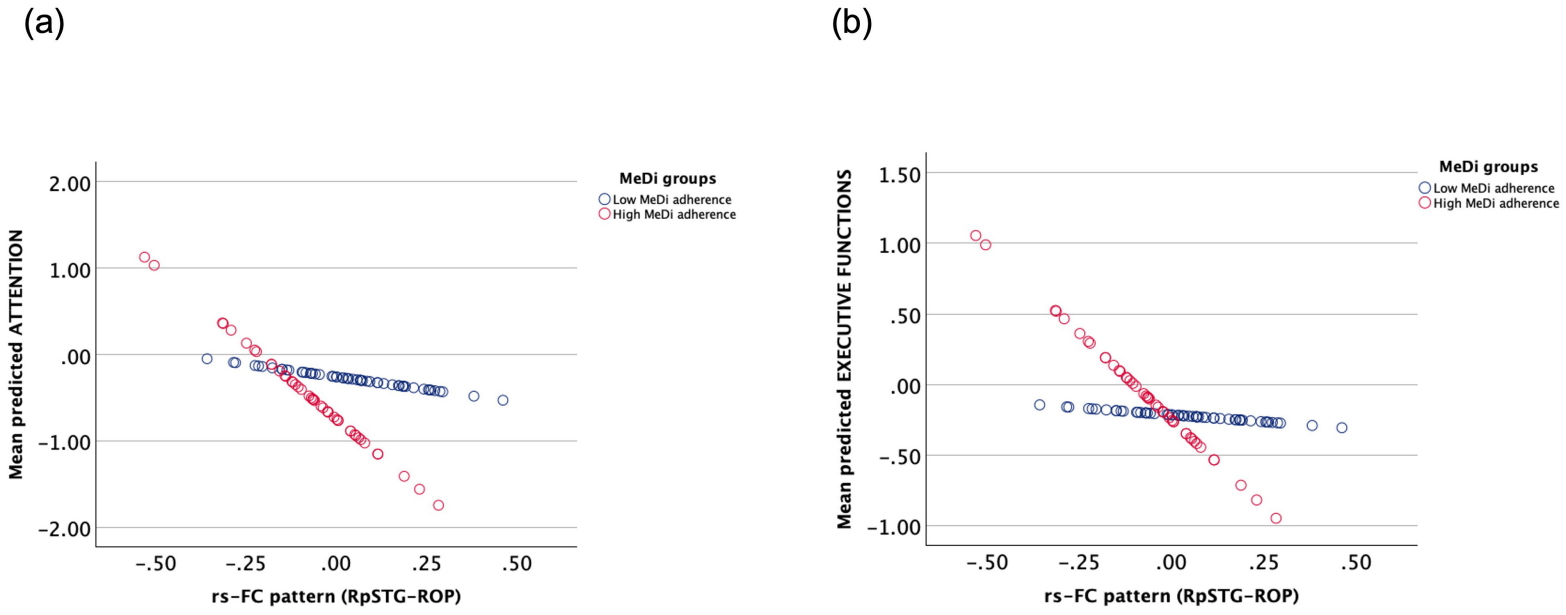
Interaction between the effect of MeDi group and rs-FC of right posterior segment of superior temporal gyrus and right occipital pole on **(A)** attention and **(B)** executive functions. MeDi groups were defined based on the median MedDietScore of the group and all participants were considered as participants with low and high adherence to MeDi. MeDi, Mediterranean diet; rs-FC, resting-state functional connectivity; RpSTG, right posterior segment of the superior temporal gyrus; ROP, right occipital pole.

We found a significant interaction between MeDi and the functional connection of right posterior superior temporal gyrus and right occipital pole on attention (p-interaction = 0.042) and executive functions (p-interaction = 0.044) (Table 3). Further analysis revealed that rsFC had a significant effect on attention (B = −3.525, *p* = 0.005) and executive functions (B = −2.461, *p* < 0.001) in the high MeDi group but not in the low MeDi group (attention: B = −0.586, *p* = 0.523; executive functions: B = −0.198, *p* = 0.783) (Figure 4). No other interactions were significant at *p* < 0.05.

**Table 3:** Functional connections related to MeDi, their associations with cognition and interactions between these functional connections and MeDi on cognition.

**Cognitive domains**	**Final model**	**Significant predictors**
Attention	*F* = 4.360, *p* = 0.015, adj. *R*^2^ = 0.065	RpSTG-ROP: B = −1.474 (−2.856, −0.091), *p* = 0.037
		MeDi^*^RpSTG-ROP: B = −0.232 (−0.454, −0.009), *p* = 0.042
Executive functions	*F* = 5.280, *p* = 0.007, adj. *R*^2^ = 0.088	RpSTG-ROP: B = −1.156 (−2.080, −0.233), *p* = 0.015
		MeDi^*^RpSTG-ROP: B = −0.154 (−0.304, −0.004), *p* = 0.044
Memory	*F* = 5.783, *p* = 0018, adj. *R*^2^ = 0.046	RpSTG-ROP: B = −1.533 (−2.797, −0.268), *p* = 0.018
Language	ns	n/a
Visuo-spatial functioning	ns	n/a

MeDi, Mediterranean diet; RpSTG, right posterior segment of the superior temporal gyrus; ROP, right occipital pole; B, unstandardized regression coefficient; CI, 95% Wald confidence interval for the unstandardized regression coefficient (lower limit, upper limit); adj, adjusted; ns, non-significant at *p* < 0.05; n/a, not applicable. For language and visuos-spatial functioning, none of the variables contributed to the final model.

## 4 Discussion

Our study examined the association between the MedDietSore; and brain FC in 105 participants without dementia. Significant associations were identified between adherence to the MeDi and anatomical regions participating in frontoparietal and temporo-occipital rs fMRI networks. In particular, the higher the MedDietScore (higher adherence to MeDi), the lower the negative FC between medial prefrontal cortex and a cluster covering part of the left postcentral gyrus and left supramarginal gyrus. On the other hand, the higher the MedDietScore, the higher the negative FC between (a) the medial visual and the right posterior segment of middle and superior temporal gyrus and (b) the right posterior segment of superior temporal gyrus and the right occipital pole. These regions are often reported as parts of functionally and/or structurally connected networks, which are known to be implicated in normal and pathological aging. Of note, we found that the FC between temporo-occipital regions (right superior temporal gyrus and right occipital pole) was associated with participants' performance on tests of attention, executive functions, and memory, whereas the association for attention and executive functions was significantly stronger for participants with high adherence to MeDi compared to participants with low adherence to MeDi.

### 4.1 Temporal inverse correlation in spontaneous BOLD fluctuations

To the best of our knowledge, this is the first study analyzing adherence to MeDi and both positive and negative correlations between anatomical regions on rs-fMRI. Previous studies focused only on positive inter-network correlations (Gaynor et al., 2022a,b) but yet did not find any significant association between MeDi and overall inter-network FC. In our study, the FC values between the different anatomical areas in all the associations with MedDietScore were negative, indicating the temporal inverse correlation in spontaneous BOLD fluctuations (anti-correlation).

While positive correlations between different brain networks may play a unifying role by combining neuronal activity that supports similar goals or representations, anti-correlations might play a distinguishing role by separating neuronal processes that support opposite goals or competing representations and by facilitate task-switching from rs conditions to task performance (Dosenbach et al., 2006; Chiong et al., 2013). Although researchers have spent significant efforts to comprehend the positive connectivity in different anatomical areas, the available studies investigating the negative connectivity values are limited (Kelly et al., 2008; Hampson et al., 2010). Anti-correlation was first identified between the task-activated brain regions and the nodes of the DMN which were deactivated during the task in normal subjects (Fox et al., 2005). The existence of inverse correlation has previously been a subject of debate in terms of artificially anti-correlations introduced by a pre-processing approach to remove noise from BOLD imaging data (global signal regression) (Aguirre et al., 1998; Desjardins et al., 2001; Macey et al., 2004; Chai et al., 2012). However, the observed anticorrelations in our study were not artifacts of the global signal regression since we applied a valid component base noise reduction method (CompCor), implemented in CONN toolbox that eliminates the above artificial effect (Behzadi et al., 2007). Of note, the temporal anti-correlation between task-positive networks engaged during task executions and task-negative networks, such as the DMN, is normally found in healthy population and it is reduced in aging and along the progression of AD (Fox et al., 2005; Dennis and Thompson, 2014; Meskaldji et al., 2016; Weiler et al., 2017; Lin et al., 2018). In line with this theoretical point of view and the existence of considerable ambiguity in the interpretation of negative correlations (Murphy et al., 2009; Chai et al., 2012), the only available studies in the literature which investigated the association between MeDi and overall inter-network FC either cross-sectionally (Gaynor et al., 2022b) or longitudinally (Gaynor et al., 2022a), excluded all negative correlation values from their analysis.

### 4.2 The association between FC and adherence to MeDi

Having established the validity of negative correlations in rs-fMRI analysis (Behzadi et al., 2007), we assume that our results have a neurobiological basis and potentially contribute to the understanding of neurophysiological mechanisms in aging, providing evidence for the role of MeDi in brain FC. Of note, we did not find any significant association between MedDietSore and participants' cognitive measures probably due to power issues or participants' characteristics. The fact that (a) MedDietSore was associated with FC but not directly to cognitive measures and (b) some of these functional connections were associated with participants' attention, executive and memory performance, and these associations were significantly stronger in participants with high adherence to MeDi compared to those with low adherence to MeDi, may indicate that MeDi potentially impact cognition through changes in FC and that rs-fMRI is a sensitive method to capture brain alterations. Changes in FC may reflect an intermediate phenotype before the emergence of cognitive changes. Previous studies have noted patterns of functional connectivity measured through rs-fMRI predict novel individuals' cognitive changes in a highly heterogeneous aging populating, highlighting that rs-fMRI may provide clinically relevant information about cognitive functions and add to the identification of markers that capture cognitive changes in normal and pathological aging (Lin et al., 2018). A similar pattern favoring our current findings was recently observed in another study focusing on MeDi, memory function, and structural connectivity (Christidi et al., 2024).

In our study, the negative correlation between occipital regions (medial visual, right occipital pole) and right temporal regions (right posterior segment of superior and middle temporal gyrus) was strengthened with higher adherence to MeDi. Of note the stronger the anticorrelation between superior temporal gyrus and occipital lobe, the higher the cognitive performance on tests of attention, executive functions, and memory. These functions are among the core cognitive functions that show alterations in normal and pathological aging (Petersen et al., 1992; Fernandez-Duque and Black, 2006; Bisiacchi et al., 2008). The FC between temporal and occipital lobes mediates in audiovisual integration processes, and the ability to combine information from the spoken message with the visual information from a speaker's face (Beauchamp et al., 2004; Noesselt et al., 2007, 2012; Uno et al., 2015). Right temporal regions are implicated in the integration of phonetic detail with talker information (Formisano et al., 2008; von Kriegstein et al., 2010; Evans and Davis, 2015; Luthra et al., 2023). Therefore, we could argue that distinct neural segregation of occipito-temporal networks may also be important to voice identity. There is also evidence that the role of the occipito-temporal network is crucial during high-order language processing. For example, superior and middle temporal and occipital anatomical regions are activated during reading while deactivation in the occipito-temporal network is associated with increased reading speed (Sun et al., 2024). In the same context, higher adherence to MeDi has been associated with higher language scores (Anastasiou et al., 2017) and better verbal abilities (Corley et al., 2013). We also noted a positive association between the MedDietScore and the FC of medial prefrontal cortex and left postcentral gyrus/anterior segment of the supramarginal gyrus; higher the adherence to MeDi was associated with reduced anti-correlation between these two anatomical regions. To the best of our knowledge, adherence to the MeDi has never been linked to negative effects on brain function. In addition, this pattern of FC was not related to any cognitive domain. Therefore, although these regions are implicated in several higher-order cognitive processes, including integration of sensory information, cognitive and motor response inhibition, word processing and phonological decision, ability to use tools, episodic memory encoding, and social cognition (Hartwigsen et al., 2010; Oberhuber et al., 2016; Lesourd et al., 2017; Christidi et al., 2018; Rubinstein et al., 2021; Friedman and Robbins, 2022), any direct interpretation of these findings would be speculative and thus future longitudinal studies are necessary to replicate our findings and provide a more plausible explanation.

In contrast to previous cross-sectional and longitudinal studies on MeDi and FC (Gaynor et al., 2022a,b) which focused only on positive correlation values and did not find any direct relation between MeDi and FC, we identified significant associations between adherence to MeDi and FC of specific anatomical regions. In their first study, Gaynor and colleagues did not find MeDi to be directly associated with rs-FC, yet they identified a moderating role on the relationship between inter-network FC and cognition, mostly fluid reasoning and to a lesser degree, Vocabulary (Gaynor et al., 2022b). Furthermore, in their longitudinal study, the same group found that the MeDi significantly moderated the effect of change in overall between-network and within-network FC on change in memory performance, suggestion that a healthy dietary pattern may protect memory function by attenuating the negative effects of alterations in FC over time (Gaynor et al., 2022a). Therefore, our findings are partially in line with the previous related studies and further highlight the role of MeDi on FC, since not only we found a direct association between MeDi and anticorrelating patterns of FC but we also support a moderating role of MeDi on the relationship between temporo-occipital FC and attention and executive functions. The mechanism by which MeDi exerts a role on cognition and brain structural and functional status has not been clearly defined, even though reduced inflammation and oxidative stress (Siervo et al., 2021), as well as protection against cardiovascular and metabolic diseases (Wu and Sun, 2017), have been proposed as potential mechanisms.

### 4.3 Strengths and limitations

Our study has several strengths. Regarding the evaluation of dietary intake, four 24-h recalls were made using the multiple-pass method (Conway et al., 2004). Moreover, each of our participants underwent thorough examinations by neurologists, and their statuses were determined through diagnostic consensus meetings and after detailed neuropsychological evaluation. Additionally, all imaging data acquisition and protocols were supervised by experienced neuroscientists. On the other hand, the present study suffers several limitations. Our study includes a relatively small sample posing power limitations and not allowing the generalizability of the results to a larger population. Due to power issues, complex models including additional covariates (e.g., lifestyle factors) or testing interaction effects (e.g., with age and/or sex) were not applied. Future studies may also examine the moderating role of other lifestyly factors, including physical activity. The participants were selected from a referral-based recruitment of a specific clinic, which may not be indicative of the population at large and may cause possible bias. Residual confounding cannot be excluded although we took into consideration important demographic confounders in the relationships explored. Another limitation is the cross-sectional design; we included baseline data from a longitudinal study and although significant, the observed findings provide associations but not causality between MedDietScore and patterns of FC.

## 5 Conclusion

Our study documented an association between MedDietScore and rs-FC in fronto-parietal and temporo-occipital regions, particularly in areas that are involved in cognitive processes altered across normal and pathological aging. The FC between superior temporal gyrus and occipital regions was associated with participants' attention, executive functions, and memory scores. Furthermore, the associations for attention and executive functions were pronounced in participants with high adherence to MeDi compared to those with low adherence to MeDi. From a clinical point of view, our findings support a favorable role of MedDietScoreon FC which may have significant clinical implications in the rapidly aging population. Rs-fMRI is also proposed as a useful tool in the emerging field of nutritional neuroscience and a candidate non-invasive biomarker of brain aging. Future longitudinal studies with a larger sample are necessary to establish causality between MedDietScoreand FC patterns and better address the long-term effects of diet on brain FC.

## Data Availability

The raw data supporting the conclusions of this article will be made available by the authors, without undue reservation.
