# Supplementary material for: Mediterranean diet and brain functional connectivity in a population without dementia
Source: Front Neuroimaging. 2024 Dec 6;3:1473399. doi: 10.3389/fnimg.2024.1473399 (PMC11659224; doi:10.3389/fnimg.2024.1473399)
Supplement: Supplementary file 1 [file Table_1.docx]

**Supplementary Table 1: Descriptive values of 11 food groups**

| **Food group** | **Mean ± std** |  | **Food group** | **Mean ± std** |
| --- | --- | --- | --- | --- |
| MDS1 | 1.95 ± 1.62 |  | MDS7 | 4.76 ± 0.90 |
| MDS2 | 1.80 ± 1.89 |  | MDS8 | 2.66 ± 1.85 |
| MDS3 | 2.62 ± 1.37 |  | MDS9 | 4.29 ± 1.24 |
| MDS4 | 2.39 ± 1.18 |  | MDS10 | 4.71 ± 0.76 |
| MDS5 | 1.43 ± 1.87 |  | MDS11 | 1.81 ± 2.41 |
| MDS6 | 1.69 ± 2.06 |  |  |  |

Notes: MDS: Mediterranean Diet Score ; std: standard deviation.
